# Rare tuberculosis in recipients of allogeneic hematopoietic stem cell transplantation successfully treated with contezolid–a typical case report and literature review

**DOI:** 10.3389/fcimb.2023.1258561

**Published:** 2023-10-16

**Authors:** Junhong Li, Zhaoxian Yu, Yingyi Jiang, Suihua Lao, Dexian Li

**Affiliations:** ^1^ Department of Critical Care Medicine, Guangzhou Chest Hospital, Guangzhou, China; ^2^ State Key Laboratory of Respiratory Disease, Guangzhou Medical University, Guangzhou, China

**Keywords:** hematopoietic stem cell transplantation, tuberculosis, antibiotic therapy, contezolid, myelosuppression

## Abstract

**Background:**

Tuberculosis (TB) is a rare but potentially devastating complication in hematopoietic stem cell transplantation (HSCT) recipients. Myelosuppression-related antibiotics should be used cautiously in patients with hematological malignancies, especially those undergoing bone marrow transplantation and receiving bone marrow suppression therapy. Although linezolid has become the recommended drug for severe TB, its hematological toxicity is still an obstacle to its clinical application. Contezolid is a new representative of oxazolidinones in clinical development, showing superior anti-infection efficacy, but there have been no reports on the treatment of post-HSCT TB.

**Case presentation:**

We reported a patient with acute lymphoblastic leukemia suffered from pulmonary TB infection after HSCT. During anti-TB treatment, the patient had a poor response to linezolid-containing regimen, and developed side effects such as gingival bleeding and thrombocytopenia, so the administration was switched to contezolid. After 15 days of continuous treatment, the patient’s platelet increased to 58×10^9^/L, and he was discharged in stable condition. During subsequent anti-TB treatment with contezolid for more than 7 months, the platelets remained stable, and no hematological adverse reactions and no symptoms of peripheral neuropathy were observed. Moreover, repeat imaging showed that the bilateral lung lesions were significantly reduced, indicating a good outcome for the patient.

**Conclusion:**

This was the first successful case of post-HSCT TB patients treated with contezolid-containing antibiotic management strategies, which exhibited remarkable efficacy and good safety in this deadly disease.

## Introduction

Hematopoietic stem cell transplantation (HSCT) recipients have severe impaired cell-mediated immune function due to the conditioning regimen used, immunosuppressive therapy, and graft-versus-host disease (GVHD). As a result, they are susceptible to bacterial, viral and fungal infections (Fan et al., 2015). These patients may also suffered from mycobacterial infection, which is life-threatening, although the incidence rate is not high, ranging from 0.0014% to 3% (Lee et al., 2014). Diagnosis of Tuberculosis (TB) in HSCT recipients can be challenging due to atypical presentation, especially when *Mycobacterium* TB (MTB) co-exists with other pathogens (Wang and Yu, 2020). Moreover, HSCT recipients often do not have a durable immune response to *Mycobacterium*, which also contributes to the increased difficulty of diagnosis. Except for this, reducing ongoing immunosuppressive therapy is recommended whenever feasible, paying attention to the risk of immune reconstitution inflammatory syndrome (Canaani et al., 2013). Notably, the reported drug-drug interactions between anti-mycobacterium drugs and hematological and immunosuppressive therapies have compounded the particularly complex management of mycobacterial infections in hematological patients (Bergeron et al., 2022).

According to the general recommendations, TB patients were treated with isoniazid, rifampicin, pyrazinamide and ethambutol for 2 months, and then treated with isoniazid and rifampicin for 4 months (Nardotto and Bollela, 2022). However, it should also be noted that the medication regimen and course of treatment for each patient need to be tailored to the specific situation of the patient, which is affected by drug toxicity, patient tolerance, drug interaction and other factors (Bergeron et al., 2022).

Oxazolidinones are a new class of synthetic antibiotics after sulfonamides and quinolones. They have attracted much attention due to their unique mechanism of action and good antibacterial activity, and there is no cross-resistance phenomenon with other drugs (Carvalhaes et al., 2020). Linezolid is the first-generation oxazolidinone drug, and has been proved to have strong anti-TB activity. Short-term use of linezolid is well tolerated, but there are still dose- and time-related adverse events such as anemia, thrombocytopenia, and peripheral neuropathy, which seriously affect the treatment process of patients (Imperial et al., 2022). Contezolid is a new generation of oxazolidone drugs with independent intellectual property rights in China, which has been approved for marketing in China in 2021. By changing the pharmacochemical conformation of linezolid, the B ring of the molecular structure of contezolid forms a non-coplanar structure with the A ring and the C ring, which enhances the binding to the bacterial target, thereby reducing mitochondrial inhibition and ultimately effectively reducing the myelosuppression (Zhao et al., 2022). Besides, the improved activity of contezoid can be largely attributed to this dramatic special change (Gordeev and Yuan, 2014). Due to the fact that critically ill infected patients often have low organ function and rapid disease progression, attention must be paid to drug interactions during multi-drug treatment. Fortunately, the unique metabolic pathway of contezoid solves this problem. It is not metabolized by the CYP liver drug enzyme system, but through the yellow monooxygenase pathway, which has little inhibition on the monoamine oxidase system, so it has no interaction with most common drugs, and can be well adapted to the combination therapy (Hoy, 2021). Consequently, contezolid has the potential to offer a promising alternative therapy for post-HSCT TB, but there was no research on treating this deadly disease.

This was the first report of successful treatment of post-HSCT TB patients with acute lymphoblastic leukemia by contezolid-containing regimen. The report of this case showed that contezolid is a new breakthrough in the treatment of this deadly disease, expanding the drug options for patients, which will also improve our understanding and treatment of TB. The study was approved by Guangzhou Chest Hospital Ethics Committee. Written informed consent was obtained from the individual for the publication of any potentially identifiable images or data included in this article. In addition, the patient and his family were aware of his condition and previous treatment history, and know that the contezolid used was new to the market, as well as the situation of other patients who have used this drug. The patient and their family agreed to try treatment with this antibiotic.

## Methods

### Bacteriological examination

For sputum culture and tracheoscopy, the sputum and bronchial fluid of patient were collected and tested in the BACTEC MGIT 960 culture detection system with Middlebrook 7H9 liquid medium (Becton, Dickinson and Company, USA).

### Molecular detection

For NGS sequencing, bronchial fluid was collected and the sequencing was performed on Illumina HiSeq2500 and NextSeq1000 instruments (Illumina). Fecal X-pert detection was performed using real-time fluorescence quantitative nucleic acid amplification detection technology (Cepheid, USA) and 7H10 solid medium was used to identify *Mycobacterium*. Fecal TB-RNA detection was performed using the Mycobacterium tuberculosis nucleic acid detection kit (Shanghai Rendu Biotechnology Co., Ltd.) on the APPLIED BIOSYSTEMS fluorescence quantitative analyzer (Applied Biosystems, USA).

### Case details

The patient, a 58-year-old male, was admitted to the hospital on November 10, 2021, due complaining of fever for 7 days. There was no family history. On November 2019, he was diagnosed with B-cell acute lymphoblastic leukemia and received multiple chemotherapy sessions, during which his bone marrow was retested as minimal residual disease-negative complete remission (MRD-negative CR). On November 26, 2020, the patient underwent allogeneic HSCT and received treatment such as leukocytosis, GVHD prophylaxis, and infection prophylaxis. On December 29, 2020, he developed thrombocytopenia and was treated with platelet transfusion after repeatedly rechecking bone marrow CR and MDR, and the results were negative and complete implantation of FISH donor. Fever occurred repeatedly in February, March, and May 2021, and after anti-infection, anti-fungal, and anti-viral treatment, the patient improved and was discharged.

**Figure 1:**
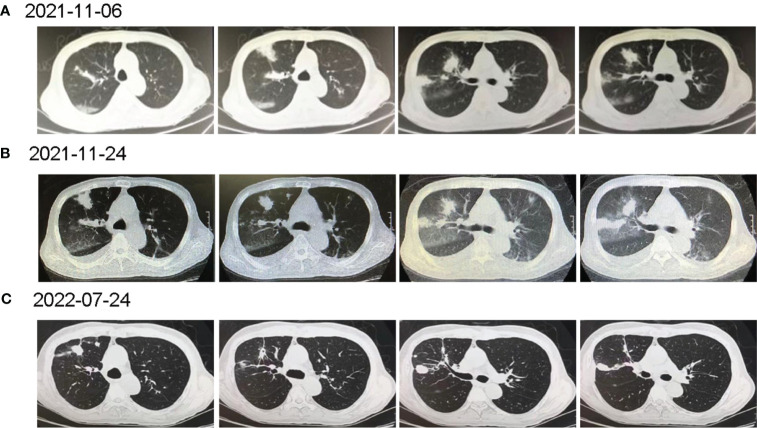
The computed tomography diagnosis of admitted patient. **(A)** Chest CT revealed multiple inflammations in both lungs, with localized pulmonary solidity and a few foci of fibroproliferation in the anterior segment of the upper lobe of the right lung; **(B)** Chest CT (15 days post-treatment) showed increased exudative lesions in both lungs and right pleural effusion; **(C)** Chest CT (8 months post-treatment) revealed that the lesions in both lungs were significantly reduced.

7 days before admission, the patient developed a fever with the highest temperature of 39.3°C. He was hospitalized again in a tertiary hospital in Guangzhou, and the chest CT showed multiple inflammations in both lungs (**Figure 1A**). He was given anti-infections such as imipenem/cilastatin, caspofungin, and liposomal amphotericin B, but he still had recurrent fevers. After completing the tracheoscopy, the bronchial fluid was found positive for MTB complex nucleic acid, and MTB was detected again in the NGS. Based on the above clinical data, the patient was considered to be post-HSCT TB, and he was admitted to our hospital after consultation.

### Transfer diagnosis

On transfer, his initial vital signs included a body temperature of 39°C, pulse was 79 beats/min, respiration was 20 beats/min, and the blood pressure was 133/84 mmHg. The patient has a clear mind, but poor spirit. Besides, the patient’s breath sounds in both lungs were clear, with no dry-wet rales, and mild edema was noted in both lower limbs. In pertinent laboratory examinations, his white blood count (WBC) was 3.42×10^9^/L, red blood cell (RBC) was 10×10^12^/L, platelets was 27×10^9^/L, lymphocyte (LY) was 0.28×10^9^/L, neutrophil ratio was 27%, procalcitonin was 0.68 ng/mL, C-reactive protein was 107.13 mg/L, serum creatinine was 135.3 μmol/L, total bilirubin was 35.79 μmol/L, and direct bilirubin was 25.43 μmol/L (**Table 1**). Sputum rapid liquid mycobacterium culture was positive, and the species was identified as MTB. Further, the stool Gene X-pert detected MTB, which was susceptible to rifampicin and the stool was positive for TB-RNA.

**Table 1:** Laboratory findings of Guangzhou Chest Hospital.

Laboratory findings			
	On admission (2021-11-10)	Discharged (2021-12-15)	Reference
White blood count (×10^9/L)	3.42	4.41	3.5-9.5
Red blood cell (×10^12/L)	2.20	2.35	4.3-5.8
Platelets (×10^9/L)	27	58	125-350
Lymphocyte (×10^9/L)	0.28	1	1.1-3.2
Neutrophil ratio (%)	87.7	56.2	40-75
Procalcitonin (ng/mL)	0.68	0.12	<0.05
C-reactive protein (mg/L)	107.13	67.88	0.00-10.00
Serum creatinine (μmol/L)	135.3	89.9	70-115
Total bilirubin (μmol/L)	35.79	6.90	5.1-19.0
Direct bilirubin (μmol/L)	25.43	3.84	1.7-6.8

### Clinical course

**Figure 2:**
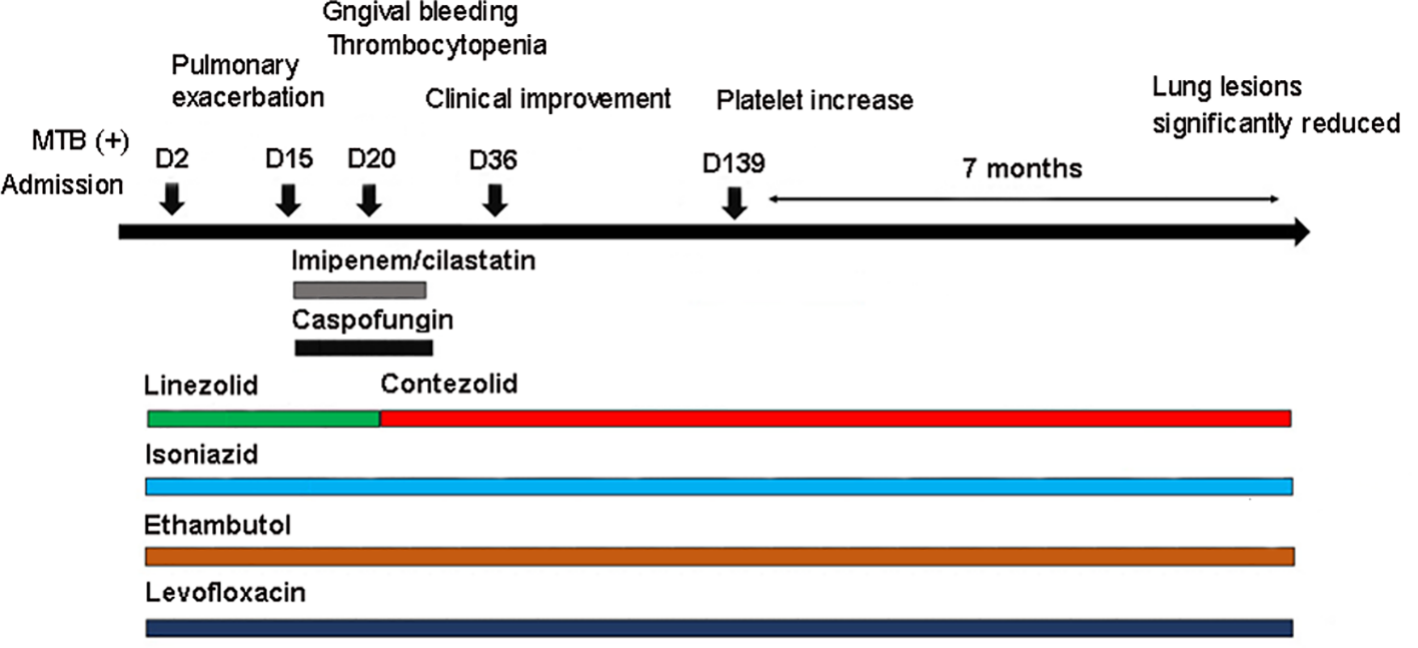
Antibiotics used in the patient and correlated changes with the progress of the clinical condition.

After admission (D1, November 10, 2021), anti-TB treatment was started under the guidance of the clinicians (**Figure 2**). Considering the patient’s long-term use of tacrolimus for anti-rejection, the presence of chronic GVHD, and poor hepatic and renal function, the regimen was formulated as isoniazid 0.6g qd, ethambutol tablets 0.75g qd, levofloxacin 0.5g qd, and linezolid 0.6g q12 h. Simultaneously, anti-fungal therapy, liver protection, platelet transfusion, and other treatments were given.

**Figure 3:**
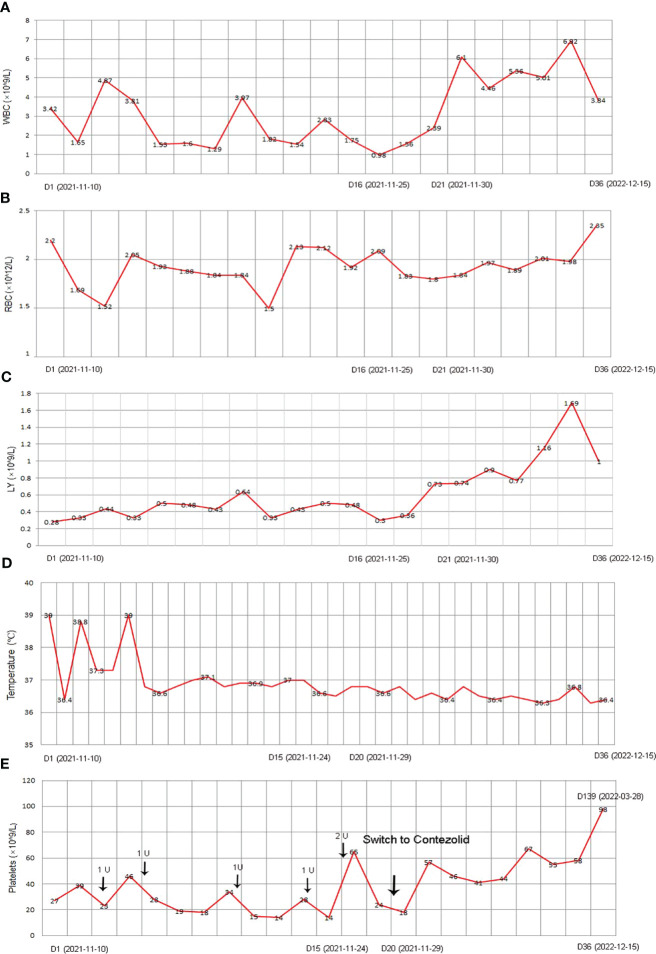
The clinical course of the patient with post-HSCT TB after corresponding treatment. **(A)** WBC; **(B)** RBC; **(C)** LY; **(D)** Temperature; **(E)** Platelet. HSCT, hematopoietic stem cell transplantation; TB, Tuberculosis; WBC, white blood count; RBC, red blood cell; LY, lymphocyte.

Under the treatment with linezolid-containing regimen, the patient’s body temperature returned to normal, and their symptoms improved compared to before (**Figure 3**). However, a repeat chest CT on D15 (November 24, 2021) showed increased exudative lesions in both lungs and right pleural effusion (**Figure 1B**). The nature of the pleural effusion puncture was exudate, and IGRAs was positive in the pleural effusion, consistent with tuberculous pleurisy. The sputum culture was positive for *Klebsiella pneumonia* and drug susceptibility suggested imipenem/cilastatin susceptibility. The next day, he was treated with imipenem/cilastatin for anti-infection and caspofungin for anti-fungal treatment. On D20 (November 29, 2021), the patient developed gingival bleeding and thrombocytopenia, and repeated platelet transfusions were ineffective (**Figure 3E**). Considering that linezolid may worsen myelosuppression, it was decided to use contezolid tablets (800 mg q12h) instead of linezolid for anti-TB treatment. On D36 (December 15, 2021), the patient’s symptoms and mental state improved, without fever, cough, sputum, shortness of breath and numbness of hands and feet. Notably, the platelets of the patients were relatively stable and showed an upward trend with an elevated platelet of 58×109/L (**Table 1**, **Figure 3E**). In view of the patient’s stable condition, he was discharged. During the whole treatment process, the patient had no discomfort, actively cooperated with the treatment, and was satisfied with the treatment results.

During follow-up on March 28, 2022 (D139), the platelet remained relatively stable at 98×109/L. The patient continued anti-TB treatment for 7 months, and the repeat chest CT (local hospital, July 24, 2022) revealed that the lesions in both lungs were significantly reduced (**Figure 1C**).

## Discussion

Clinically, fever and pneumonia are two typical characteristics of infection. 50% of fever patients cannot find clear pathogenic evidence, so empirical anti-infection treatment must be initiated first. It is particularly important to point out that when sufficient treatment courses and intensity of antifungal therapy are not effective, exclusion diagnosis of TB should be carried out (Abdullahi et al., 2021). The gold standard for diagnosing TB is to isolate MTB through culture. Among them, liquid culture is considered to be the standard method, as it has better separation quality compared to solid culture. Moreover, the introduction of this technology has greatly improved diagnosis, reducing the detection time to 10-14 days, while traditional culture requires several weeks (Molicotti et al., 2014). Encouragingly, molecular tests for MTB continue to change the landscape of TB diagnosis. Most molecular methods involving nucleic acid amplification directly detect MTB-DNA, with higher sensitivity and specificity (Bergeron et al., 2022) For X-pert, it can not only determine whether there is TB, but also whether it is rifampicin resistance. In this case, rifampicin-susceptible TB in HSCT recipients was confirmed by rapid liquid culture and multiple molecular diagnostic techniques, which deepened the previous diagnostic evidence and provided the direction for subsequent medication.

The recommended treatment for TB is isoniazid or rifampicin alone or in combination due to hepatotoxicity associated with TB drugs and drug interactions. However, rifampicin induces liver mitochondrial enzymes that reduce serum cyclosporin A levels and may therefore aggravate GVHD (Wasko et al., 2017). In addition, rifampicin was more likely to have adverse reactions when there was liver disease or when it was combined with isoniazid (Yang et al., 2022). Considering the long-term use of tacrolimus for anti-rejection, chronic GVHD and poor liver and kidney functions, rifampicin was not administered, although sensitive to rifampicin. Within mind that the excellent antibiotic action of oxazolidinones in severe infection, clinical administration focused on these drugs. For Linezolid, it has been demonstrated in multiple studies to have a high concentration in alveolar tissue with protein binding rate so as to be a potential drug for TB patients (Wei and Zhang, 2022). Therefore, linezolid plus isoniazid, ethambutol and levofloxacin anti-TB program was administrated instead of isoniazid and rifampicin. Remarkably, after the corresponding treatment, the patient’s condition was brought under control and his temperature returned to normal. However, 14 days of post-treatment, increased bilateral lung exudation and a new pleural effusion on the right side were observed. Moreover, during treatment, the patient suffered from gingival bleeding, thrombocytopenia and other symptoms, and the situation did not improve after several platelet transfusions, indicating that the patient had a poor response to anti-TB regimen containing linezolid. We speculated that this may be related to the aggravation of myelosuppression by linezolid. In an open-label, single-group study, 81% of patients developed linezolid-induced peripheral neuropathy and 48% developed linezolid-induced bone marrow suppression (Conradie et al., 2020). Notably, this patient belongs to a high-risk population and is more prone to hematological adverse reactions than other patient groups (Averbuch et al., 2013). This may explain why the patient was poorly treated. However, considering that the body temperature was stable, linezolid was not considered to be completely ineffective. The individual differences, different levels of disease severity, and heterogeneous drug resistance may affect drug efficacy and choice.

Despite the high success rate of linezolid reported, the protocol cannot be used as a routine treatment worldwide due to the worrying safety profile, and more evidence on safety needs to be gathered in future studies. Thus, linezolid was discontinued and a newer, safer oxazolidinone drug was sought as an alternative. In an *in vitro* study, the intracellular bactericidal activity of contezolid against MTB was much higher than that of linezolid, suggesting that contezolid is an ideal choice for the treatment of TB (Wang et al., 2022). In an elderly patient with tuberculous pleurisy, the anti-TB treatment with contezolid instead of linezolid also showed a good effect (Kang et al., 2022). In this case, approximately 7 months after the treatment strategy was switched to the contezolid regimen, the patient’s symptoms improved with normal temperature, and bilateral lung lesions were significantly reduced compared to previous treatments, indicating that the contezolid-containing regimen exerts a good anti-TB effect. The accumulated national and international data also provides strong evidence for the safe application of contezolid. In the Phase I clinical trial conducted in Australia, no volunteers experienced hematological or lymphatic adverse events related to contezolid. However, 3 subjects developed thrombocytopenia or leukopenia due to linezolid, and 1 subject withdrew early due to significant hematology-related toxicity (Eckburg et al., 2017). In a Phase III clinical trial conducted in China, it was found that there were significantly fewer serologic abnormalities in the contezolid group than in the linezolid d group (Zhao et al., 2022). This was consistent with the safety profile of contezolid observed in our study. After the antibiotic was adjusted to contezolid, the patient’s platelet count increased to 58×10^9^/L. After 8 months of treatment, platelets remained relatively stable, and no hematology adverse events were observed. Moreover, there were no symptoms related to peripheral neuropathy, such as numbness in hands and feet. Importantly, it should be noted that throughout the entire treatment process, the patient showed no discomfort and satisfaction with the treatment with contezolid, and strongly believed that contezolid could play an irreplaceable role in the field of the disease. Collectively, the contezolid-containing regimen has demonstrated excellent efficacy and safety in post-HSTC TB.

There are some limitations in the manuscript. First, the patient was treated with contezolid and other antibiotics at the same time, so it is difficult to explain the therapeutic effect of a single antibiotic. Second, there was only one patient in this study, and the results for a single patient are not generalizable because the medication regimen and course of treatment for each patient needs to be tailored to the individual patient. However, the successful treatment of this rare case showed that the potential for contezolid is enormous, expanding the medication options for patients and improving our understanding of TB. Further, contezolid needs to be tested in a larger population with increased follow-up time to obtain more convincing and longer-term data. Besides, In addition, given the rarity of this post-HSCT TB, parallel *in vivo* and *in vitro* studies will be conducted to increase understanding of contezolid.

## Conclusion

This was a successful exploration of contezolid in the treatment of TB infection after HSCT, indicating that the emergence of the contezolid could be a new weapon in the fight against TB and will bring more benefits to TB patients. However, large population and long-term follow-up studies are needed to provide more convincing evidence.

## Data availability statement

The original contributions presented in the study are included in the article/supplementary material. Further inquiries can be directed to the corresponding author.

## Ethics statement

The studies involving humans were approved by ethics committee of Guangzhou Chest Hospital. The studies were conducted in accordance with the local legislation and institutional requirements. The participants provided their written informed consent to participate in this study. Written informed consent was obtained from the individual for the publication of any potentially identifiable images or data included in this article.

## Author contributions

JL: Conceptualization, Data curation, Writing – original draft, Writing – review & editing. ZY: Data curation, Formal Analysis, Methodology, Writing – review & editing. YJ: Formal Analysis, Project administration, Writing – review & editing. SL: Formal Analysis, Methodology, Project administration, Writing – review & editing. DL: Conceptualization, Data curation, Methodology, Supervision, Writing – original draft, Writing – review & editing.
